# Supplementary material for: Serotype distribution and antimicrobial resistance of human Salmonella enterica in Bangui, Central African Republic, from 2004 to 2013
Source: PLoS Negl Trop Dis. 2019 Dec 2;13(12):e0007917. doi: 10.1371/journal.pntd.0007917 (PMC6907862; doi:10.1371/journal.pntd.0007917)
Supplement: Attachment — Submitted filename: Breurec_Salmonella_Reviewer 2.docx [file pntd.0007917.s001.docx]

Prof. Sébastien BREUREC	September, 2^th^ September 2019

Institut Pasteur

To:	Elsio Wunder Jr

Deputy Editor
PLOS Neglected Tropical Diseases

Dear Colleague,

We are grateful for your consideration of this manuscript, and we also very much appreciate your suggestions, which have been very helpful in improving the manuscript. We also thank the reviewers for their careful reading of our text.

All the comments we received on this study have been taken into account in improving the quality of the article, and we present our reply to each of them separately. Two versions of the manuscript are enclosed, one where all the changes have been underlined, and another version without any marks.

We hope that these changes to the manuscript will facilitate the decision to publish this study in your journal. We have made an effort to take into account the interesting suggestions proposed by the reviewers. In any case, we are open to consideration of any further comment on our answers.

Best regards

Sébastien BREUREC

**Reviewer's Responses to Questions**

**Methods**

**Reviewer #1**:

This study, including a large number of strains describe the epidemiology of *Salmonella* infections in CAR between 2004 and 2013. His strength is to be interested in strain genetics. Furthermore, data concerning the antimicrobial resistance in this part of the world are substancial.
The manuscript is clear and easy to read and objectives are clearly defined.

**Reviewer #2:**

Line 99: Please clarify what is meant by "no additional specimens were collected for this study". At this status of the manuscript it is not clear/known to the reader which samples actually were collected.

**Answer :** We agree with the rewiever. *We remove this sentence because it is unclear.* This study is retrospective study, then, by definition 'non-interventional'.

We add the following sentence (line 99): « This retrospective study is based on a retrospective data collection issued from the routine bacteriological diagnostic activity within the private clinical laboratory located at Institut Pasteur in Bangui. »

L101: To which healthcare facility are authors referring to?

**Answer :** The term « healthcare facility » is not appropriate. We modify by private clinical laboratory (see above – line 99).

Which type of patients (including their eligibility criteria) and infectious/chronic diseases are under the routine surveillance mentioned?

**Answer :**

Many thanks for these comments. *We removed the following sentence because it is unclear* : « This study was therefore part of the routine surveillance measures within this healthcare facility. »

The laboratory of the Institut Pasteur is a major clinical diagnostic laboratory in Bangui. Almost 150 patients are cared for each day, most of them suffering from an infectious disease. On average, 20 stool bacteriological cultures from patients suffering from diarrhea are performed each day. Between 2004 and 2013, all patients with positive biological sample cultures to *Salmonella enterica* were included in this study.

Two new sentences have been added to answer this question:

- Section « Material and methods » (line 99): « This retrospective study is based on a retrospective data collection issued from the routine bacteriological diagnostic activity within the private clinical laboratory located at Institut Pasteur in Bangui. »

- Section « Results » (line 108): « All non-duplicate S*. enterica* clinical isolates collected between January 2004 and December 2013 were included.

L111: From which biological samples were the isolates detected ?

**Answer :**

The S*. enterica* isolates were detected from stools, blood, urines, cerebrospinal fluids, wounds and from one pleural fluid.

We modified the following sentence in the section « results » (line 193): « Isolates were from stool (n=452, 77%), blood (n=97, 17%), urine (n=14, 2%), cerebrospinal fluid (n=13, 2%), wound (n=5, 1%) and pleural fluid (n=1) samples. »

L114-117: Which antibiotic concentration were tested?

**Answer :**

As indicated by the reviewer, we modified the following sentence (line 119): « Susceptibility to amoxicillin (20 μg), amoxicillin-clavulanic acid (20-10 μg), ticarcillin (75 μg), cefalotin (30 μg), cefoxitin (30 μg), cefotaxime (5 μg), ceftazidime (10 μg), amikacin (30 μg), streptomycin (10 UI), tobramycin (10 μg), gentamicin (10 μg), spectinomycin (10 μg), nalidixic acid (30 μg), ciprofloxacin (5 μg), chloramphenicol (30 μg), sulphonamides (200 μg), cotrimoxazole (1.25-23.75 μg) and tetracycline (30 μg) was tested by the disc diffusion method on Mueller-Hinton agar (Bio-Rad), and production of ESBL was detected by the double-disc synergy (DDS) test according to the 2018 guidelines of CA-SFM/EUCAST (http://www.sfm-microbiologie.org)”

**Results**


**Reviewer #1:**

The analysis are in accord with the plan and the results are clearly presented and easy to read. 
- The Table 2 could be enlarged
- It would be interesting to detail the various serotypes of Salmonella responsible for bacteremia and meningitis

**Answer** : Many thanks for these comments. It is difficult to enlarge the table 2 because it contains so many data. However, we specify the different serotypes associated with bacteremia and meningitis in the section « results ».

We add the following sentences (line 193): « Isolates were from stool (n=452, 77%), blood (n=97, 17%), urine (n=14, 2%), cerebrospinal fluid (n=13, 2%), wound (n=5, 1%) and pleural fluid (n=1) sample. Of the blood isolates, 77 (79%) were NTS, and Typhimurium (n=49, 64%% of the isolates) and Enteritidis (n=15, 19%) were the predominant serotypes. The other NTS serotypes were Dublin (n=6), Stanleyville (n=3), Brancaster (n=1), Hartford (n=1), Mikawashima (n=1) and Saintpaul (n=1). Three blood isolates were not typeables and 17 belonged to serotype Typhi. A total of 13 patients with meningitis were reported during the study period, and Typhimurium was involved in 9 cases (69%). The other serotypes were Enteritidis (n=1), Poona (n=1), Sainpaul (n=1) and Typhi (n=1).

- Can the authors specify in which site (stool, blood or other ) the Salmonella producing ESBL was isolated?

**Answer** : The Salmonella producing ESBL was isolated from blood.

We modified the following sentence (line 224): « The blood Typhimurium isolate S1027072 was also resistant to the beta-lactams tested (ceftazidime: MIC 32 mg/L, ceftriaxone: MIC >256 mg/L), except for cefoxitin and imipenem.»

**Reviewer #2:**

L 183-184: So, 9/13 patients with meningitis had a co-infection with S. Typhimurium?

**Answer:** The patients had meningitis due to *Salmonella enterica*. It’s not a co-infection.

We modify the following sentence (line 201): “A total of 13 cases of meningitis due to *Salmonella enterica* were reported during the study period, and Typhimurium was involved in 9 cases (69%). The other serotypes were Enteritidis (n=1), Poona (n=1), Sainpaul (n=1) and Typhi (n=1). »

L 193: Please clarify what is meant by low resistance.

**Answer:** Many thanks for these comments. It means that the prevalence of resistance to a given antibiotic was less or equal to 2%: Amoxicillin-clavulanic a. (2%), cefalotin (1%), cefoxitin (0%), cefotaxime (0%), amikacin (0%), gentamicin (1%), nalidixic acid (2%) and ciprofloxacin (0%)

We modified (line 215): « Overall, the 582 *S. enterica* isolates displayed a low level of resistance (≤ 2%) to all antibiotics, except those used as traditional first-line drugs: tetracycline (18%), chloramphenicol (30%), cotrimoxazole (39%) and amoxicillin (40%) (Table 3). »

Were the isolates tested resistant to the antibiotics tested or did they possibly show some borderline/intermediate patterns?

**Answer:** Strains with intermediate patterns were not merged with the resistant ones for the analysis of the data.

We add the following sentence in the section “Materials and methods” (line 128): « Isolates of intermediate susceptibility were not classified with the resistant ones for the analysis of the data ».

Which standards were used as a reference to classify isolates based on their susceptibility/resistant patterns?

**Answer:** We add the following sentence (line 126): « Isolates were classified as resistant, intermediate or susceptible according to the 2018 guidelines of CA-SFM/EUCAST (http://www.sfm-microbiologie.org). »

**Answer to the editor**

With regards to the use of « unpublished » 2 times in the manuscript on Table 1, we have deleted the corresponding column. All the nucleotidic sequences used in our MS were deposited in Genbank (see accessions numbers) and are freely available. However, for two of these sequences, no published article had included these sequences. That's why we used the term "unpublished" in the table 1. We agree with the reviewer that it was unclear. In the column “accession number”, the readers will have the accession number to access to the nucleotide sequence, and then, to the corresponding publication if available.

Thank you very much for the constructive comments that have certainly contributed to improve this MS.
