# Supplementary material for: Serotype distribution and antimicrobial resistance of human Salmonella enterica in Bangui, Central African Republic, from 2004 to 2013
Source: PLoS Negl Trop Dis. 2019 Dec 2;13(12):e0007917. doi: 10.1371/journal.pntd.0007917 (PMC6907862; doi:10.1371/journal.pntd.0007917)
Supplement: Attachment — Submitted filename: Breurec_Salmonella_Reviewer 3.docx [file pntd.0007917.s002.docx]

Prof. Sébastien BREUREC	September, 1^th^ October 2019

Institut Pasteur

To:	Elsio Wunder Jr

Deputy Editor
PLOS Neglected Tropical Diseases

Dear Colleague,

We are grateful for your consideration of this manuscript, and we also very much appreciate the suggestions of the reviewer 3, which have been very helpful in improving the manuscript.

All the comments we received on this study have been taken into account in improving the quality of the article, and we present our reply to each of them separately. Two versions of the manuscript are enclosed, one where all the changes have been underlined, and another version without any marks.

We hope that these changes to the manuscript will facilitate the decision to publish this study in your journal. We have made an effort to take into account the interesting suggestions proposed by the reviewer 3. In any case, we are open to consideration of any further comment on our answers.

Best regards

Sébastien BREUREC

**Reviewer's Responses to Questions**

**Methods**

**Reviewer #1**:

The manuscript was corrected according to the reviewer's comments and can now be accepted

**Reviewer #2:**

No further comments.

**Reviewer #3:**

This a descriptive microbiological analysis of routine *Salmonella* isolates from an admittedly very understudied setting in the CAR with then some more detailed information on a single ESBL isolate. While the study thus provides some new data on *Salmonella* in this geographic area, it also suffers from a number of major issues

1) There is no clear testable hypothesis stated. They provide a descriptive analysis of routinely collected isolates from a single clinical microbiology lab.

We agree with the rewiever.

We modify the text as following (line 95): “The study’s primary objective was to report on the prevalence of resistance to antibiotics in *S. enterica* strains isolated from patients in CAR between 2004 and 2013 to determine whether recommendations for first-line empirical treatment based on C3G and fluoroquinolones are relevant. Secondary objectives were i) to investigate the genetic background of serotype Typhimurium to determine whether ST313 is the primary cause of invasive salmonellosis, iii) to determine the genetic basis for resistance to C3G using whole genome sequencing.”

2) A description of how the samples came into the Pasteur lab is missing. Is the Institut Pasteur lab associated with a health clinic, hospital, private GPs?

**Answer :**

Diagnostic laboratories are poorly resourced in CAR. The medical laboratory of the Institut Pasteur is the major clinical diagnostic laboratory in Bangui. It caters to patients from private health sector and receives also samples from hospitals and other clinical laboratories. Almost 150 patients are cared for each day, most of them suffering from an infectious disease. On average, 20 stool bacteriological cultures from patients suffering from diarrhea are performed each day.

We add the following sentences.

1- Line 105: « This retrospective study is based on a retrospective data collection issued from the routine bacteriological diagnostic activity within the private clinical laboratory located at Institut Pasteur in Bangui. “
2- Line 122: “ Our private clinical laboratory, the major one in the capital, performed biological analysis on specimens from its patients but also on samples from other hospitals and clinical laboratories »

Did some isolates derive from the mentioned case-control study conducted in 2011-13?

If yes, how many isolates from each "source"? What type of cases (invasive, stool) are these isolates most likely to be representative of?

**Answer** : A total of 21 isolates derived from the mentioned case-control study conducted in 2011-13 were included in this study, 13 from cases and 8 from controls. All *Salmonella* isolates from cases were only isolated from stools.

We modify the following sentence (line 124): « All non-duplicate *S. enterica* clinical isolates collected between January 2004 and December 2013 were included, encompassing those previously described [4].”

3)  Given that the title mentions "surveillance", one would at least expect a summary epidemiological analysis including age distribution & sex of cases, in addition to providing microbiological data like serotyping and antibiotic resistance testing on non-duplicate isolates. Please revise title to include "serotyping", "antimicrobial resistance" and "human" isolates

**Answer** : We thank the reviewer and we are totally agree.

The title was changed as following: « Serotype distribution and antimicrobial resistance of human *Salmonella enterica* in Bangui, Central African Republic, from 2004 to 2013 ».

4) The data collection period and the typing technique CRISPOL used are starting to be dated. Would it not be possible to extend the analysis to isolates also from the last 5 years, 2014-2018 and also provide more WGS results if possible. The authors need to say at least whether Salmonella "surveillance" is still ongoing now or was discontinued after 2013.

**Answer** : Since 2013, the CAR faced an ongoing civil war, plunging the country into a chaotic state of violence and an ensuing humanitarian crisis. The clinical laboratory of the Institut Pasteur in Bangui has been closed for many weeks in 2013. This project was supported by the WHO Laboratory twinning initiative “Improvement of diagnostic and surveillance capacity for *Salmonella* in the Central African Republic”. Currently, the grant has ended and we no longer have the financial means to carry out such a study. I left Bangui in 2014, nobody was able locally to ensure the continuity of the surveillance and this realy a very hard work (see answer below, section : “Editorial and Data Presentation”). All these elements explained why the surveillance was discontinued since 2013. The data presented here begins to date but they are unique in Central Africa. They are particularly important, given the difficulty of carrying out such studies in countries with inadequate health-care systems. However, thanks to the strong link between the Institut Pasteur International Network (RIIP) and the French NRC held at Institut Pasteur Paris in France, a WGS approach-study could be coordinated when the lab-surveillance capacities will be back locally.

We add the following sentence (line 319): « Since 2013, the CAR has faced an ongoing civil war, plunging the country into a chaotic state of violence and an ensuing humanitarian crisis. The surveillance of *Salmonella enterica* isolates at the Institut Pasteur in Bangui was discontinued but a WGS approach-study could be coordinated when the laboratory-surveillance capacities will be strengthened locally. »

5) The sample size of isolates from 2013 in table 2 is rather small compared to other years (and the serotype distribution is quite different to previous years - was S Liverpool really the top serovar?). Reasons for this needs to be at least discussed in the manuscript.

**Answer** :

A low number of *Salmonella* isolates was collected in 2013 because of the economic and political crisis that occurred, further restricting patient access to health-care facilities. Since 2013, the CAR faced an ongoing civil war. The capital, Bangui, was seized by the rebels in March 2013, plunging Bangui into a chaotic state of violence and an ensuing humanitarian crisis. Our clinical laboratory was closed for many weeks in 2013. Since the outbreak of renewed conflict in 2013, thousands of people have been killed and nearly 575,000 refugees have been displaced, the majority of whom fled to neighboring Cameroon and the Democratic Republic of Congo. (see https://en.wikipedia.org/wiki/Central_African_Republic_Civil_War_(2012%E2%80%93present)#Atrocities).

It is surprising that Typhimurium and Enteritidis, the two most frequently recovered serovars every year, were not recoverd in 2013. This is probably the consequence of the low number of isolates collected in 2013 (n=15) compared to those found each year.

We modify the following sentence (line 313) : « In this study of 582 non-duplicate *S. enterica* isolates investigated between 2004 and 2013, Typhimurium and Enteritidis were the two most frequently recovered NTS serotypes, in accordance with numerous other studies in sub-Saharan Africa [1-3, 18-23]. Surprisingly, they were not found in 2013, probably due to the small number of *Salmonella* isolates collected this year. Indeed, significant differences in the numbers of organisms collected were observed by year, due to the economic and political crisis during the study period, which further restricted patient access to health care facilities. »

6) Randomization procedure for selecting *S.* Typhimurium isolates needs to be explained more.

We thank the reviewer for his comment.

**Answer :** We add the following sentence (line 250) : « CRISPOL typing was performed on a set of 68 *S.* Typhimurium isolates, including S1027072. They were chosen using a random number table and corresponded to around 38% of the isolates collected every year.”

7) It is not clear where the various lab testing done, was all serotyping and AMR testing done in Paris at the end of the study period ?

Identification, antimicrobial susceptibility testing, conjugation experiment but also preliminary serotyping was performed at our private clinical laboratory located at Institut Pasteur in Bangui. The complete serotyping of *Salmonella* isolates as well as WGS of the ESBL isolate and CRISPOL typing were performed at the Institut Pasteur in Paris.

We modify these sentences :

1- line 131 : « The strains were serotyped on the basis of somatic O and phase 1 and phase 2 flagellar antigens by agglutination tests with antisera (Bio-Rad, Marnes-La-Coquette, France), as specified in the White-Kauffmann-Le Minor scheme (preliminary serotyping at the Institut Pasteur in Bangui, complete serotyping at the Institut Pasteur in Paris)”.
2- Line 150 : Total DNA was extracted with the Instagene kit (Bio-Rad, Marnes-la-Coquette, France). CRISPOL typing and MLST was performed on the Luminex platform (Luminex Coporation, Austin, TX, USA) at the Institut Pasteur in Paris, as previously described [6].
3- Line 153 : « WGS was performed on the ESBL-producing S. Typhimurium isolate S1027072 identified in this study and on three Salmonella isolates (serotypes Grumpensis, Havana and Telelkebir) recovered from West Africa in 2007 and 2008 and previously described, at the Institut Pasteur in Paris »

8) No description was given of how the MLST type (ST313) was obtained. I guess that the authors assumed that the CT28 is indicative of ST313, rather than having conducted MLST, i.e the ST was "deduced" based on results from a previous study in the Democratic Republic in Congo. This needs to be made clearer in methods. I recommend that authors change wording in abstract, replacing "associated with" by "indicative of". One of the problems with this approach is that the correspondence between CT28 and ST313 might be local to the Congo area. Ideally at least some CT28 S. Typhimurium isolates should be sequenced by WGS to make sure that they also correspond to ST313 in the CAR.

**Answer :** Many thanks for these comments.

We add the following sentence (line 257): « To confirm the association of ST313 to the CT28 group, MLST was performed on 10 isolates: 7 assigned to CT28, one to CT698 and one to CT526. All the isolates belonged to ST313. In addition, the ESBL-*S*. Typhimurium belonging to CT28 by CRISPOL typing was assigned to ST313 by WGS. All these data confirmed the association between CT28 group and ST313 in *Salmonella* isolates from CAR.»

For the ESBL isolate  submitted to WGS, the authors should also individually report for completeness the full antibiotic resistance profile,  the ST, as well as any non-ESBL resistance genes or mutations, for example using ResFinder or CARD.

We add the following sentence:

1- Line 243: « The blood Typhimurium isolate S1027072 was also resistant to the beta-lactams tested (ceftazidime: MIC 32 mg/L, ceftriaxone: MIC >256 mg/L), except for cefoxitin and imipenem, to gentamicin, to chloramphenicol, cotrimoxazole, and tetracyclin, and susceptible to amikacin, nalidixic acid, and ciprofloxacin. »
2- Line 257: « In addition, the ESBL-*S*. typhimurium belonging to CT28 by CRISPOL typing was assigned to ST313 by WGS. »
3- Line 290: “Both plasmids encode: (i) resistance to heavy-metal ions, including mercury (*mer* and *tni* genes), tellurite (*ter* genes), arsenic (*ars* genes), copper (*cusS* and *pcoE* genes), nickel and cobalt (*rcnR* and *rcnA* genes), and to tellurite utilization genes (*tel* genes); and (ii) resistance to antimicrobials, including aminoglycosides (*aac*(6')-Ib), streptomycin (*aadA1*, *strA* and *strB*), beta-lactams (*bla*_OXA-30_, *bla*_TEM-1_ and *bla*_CTX-M-15_), chloramphenicol (*catA1* and *catB3*), trimethoprim (*dfrA14*), sulfonamides (*sulI* and *sulII*), gentamicin (*aacC3*) and tunicamycin (*tmrB*), most of which are in a class 1 integron. Genes for conjugative transfer were also identified. The transferability of the plasmid was confirmed in the conjugation experiment. We did not find any spontaneous chromosomal mutations associated with resistance to antibiotics.”

**Answer:**

**Results

Reviewer #1:**

The manuscript was corrected according to the reviewer's comments and can now be accepted
 **Reviewer #2:**

No further comments.

**Reviewer #3:**

In table 1, it's not clear why the size information of the plasmid is not given for strains in lines 2 to 5 - is it because it is not a full assembly?

**Answer**: Yes it is not a full assembly. We add a footnote in the table 1.

The authors could report the size of the de novo assembly and then indicate as a footnote, that it might not be complete.

**Answer**: See table 1

In most developed countries, resistance patterns are strongly associated with serotype, typically higher for *S.* Typhimurium and Typhi than for other serovars. The authors do not provide any detailed AMR data for serovars other than S. typhimurium, like S. Enteritidis and S. Typhi which are also quite common. A new table should be added with showing most frequent AMR profiles for most frequent serotypes, or at least mention most most frequent AMR profiles for most frequent serovars in the text.

Answer : Thanks for the comment. This data are missing.

We add the following sentence (line 233): « The most frequent phenotype of resistance in serotypes Enteritidis (22/64, 34%) and Typhi (9/38, 24%) was susceptibility to all the beta-lactams tested, except for amoxicillin and ticarcillin, to aminoglycosides, to fluoroquinolones, and resistance to chloramphenicol, cotrimoxazole and tetracyclin.”

In table 4, for the footnotes a & b, need to add citation to paper showing the association with ST313.

**Answer :** The association of CT28 with ST313 was demonstrated for isolates from CAR.

**Conclusions**

**Reviewer #1:**

The manuscript was corrected according to the reviewer's comments and can now be accepted
 **Reviewer #2:**

No further comments.

**Reviewer #3 :**

The authors interpretation that one isolate of 582  (0.2%) being an ESBL is a "major" concern is debatable. One could also sonclude that ESBL in Salmonella in this setting are quite rare, and probably  rarer than other Enterobacteriacea in other African countries. Consider replacing "major concern" by "concern".
Line 295 the authors write that clinical features are unknown and they say in the same sentence that they reported diarrhea. This doesn't make sense. Either they have (some of) this data or they don't.

Line 300 the authors mention the "emergence" of MDR ST313 which suggests a change in frequency over time. The data presented herein does not support such a changing trend over time, so authors should consider replacing "emergence" with "substantial prevalence of". Also the last sentence about more frequent" is quite speculative as the data presented in this study does not show any increase over time.

Answer: We thank the reviewer and we modified the texte according to its comments.

1- Line 341: “Our data also confirm the substantial prevalence of multi-drug-resistant S. Typhimurium ST313”
2- This sentence was deleted (line 320): “Although the clinical features of the patients are unknown, most of those attending the Institut Pasteur for bacteriological stool analysis only reported diarrhea.”
3- Line 373: “Although the prevalence of ESBL-producing *Salmonella* *enterica* isolates was very low, the wide distribution of a single IncHI2 plasmid scaffold among *Salmonella* serotypes in sub-Saharan Africa, the origin of dissemination of the *bla*_CTX-M-15_ gene, is of concern. “

**Editorial and Data Presentation**

Reviewer #1: The manuscript was corrected according to the reviewer's comments and can now be accepted

Reviewer #2: No further comments.

Reviewer #3: Acknowledgments: it's not usual to thank technicians for doing what appears to be "routine" work.

**Answer:** We agree that serotyping appears to be “routine” work. The Institut Pasteur in Bangui was closed for several weeks in 2013 due to the political crisis and violence. All bacteria identified for diagnosis in our medical laboratory are routinely stored at -80 ° C. During the closed periods in 2013, we searched in freezers at -80 ° C all *Salmonella* isolates kept. Then, we performed preliminary serotyping. In addition, all the data concerning the susceptibility to antibiotics were extracted from our Laboratory Information Management System. It was a hard job, especially considering the conditions prevailing in Bangui. All the isolates were then sent to Paris for complete serotyping. It was also a huge job since it was realized in addition to the routine work already done in the laboratory. It took a long time. For all the reasons, I would like to thank all the technicians involved in this work.

Thank you very much for the constructive comments that have certainly contributed to improve this MS.

Pr Sébastien BREUREC
